# The hypertrophic amygdala shape associated with anxiety in patients with primary dysmenorrhea during pain-free phase: insight from surface-based shape analysis

**DOI:** 10.1007/s11682-022-00664-3

**Published:** 2022-07-24

**Authors:** Siyi Yu, Wei Wei, Liying Liu, Xiaoli Guo, Zhifu Shen, Jin Tian, Fang Zeng, Fanrong Liang, Jie Yang

**Affiliations:** 1 Brain Research Center, Acupuncture and Tuina School, Chengdu University of Traditional Chinese Medicine, 37 Shierqiao Road, Chengdu, China; 2 North Sichuan Medical College, Nanchong, China

**Keywords:** Primary dysmenorrhea, Morphometry, Amygdala, Shape analysis, Anxiety

## Abstract

**Background:**

Primary dysmenorrhea (PDM) is highly associated with mood symptoms. However, the neuropathology of these comorbidities is unclear. In the present study, we aimed to investigate the structural changes in the amygdala of patients with PDM during the pain-free phase using a surface-based shape analysis.

**Methods:**

Forty-three PDM patients and forty healthy controls were recruited in the study, and all participants underwent structural magnetic resonance imaging scans during their periovulatory phase. FMRIB’s Integrated Registration and Segmentation Tool (FIRST) was employed to assess the subcortical volumetric and surface alterations in patients with PDM. Moreover, correlation and mediation analyses were used to detect the clinical significance of the subcortical morphometry alteration.

**Results:**

PDM patients showed hypertrophic alteration of the amygdala in the left superficial nuclei and right basolateral and superficial nuclei but not for the whole amygdala volume. The hypertrophic amygdala was associated with disease duration, pain severity and anxiety symptoms during the menstrual period. Furthermore, the hypertrophic left amygdala could mediate the association between disease duration and anxiety severity.

**Conclusions:**

The results of the current study demonstrated that the localized amygdala shape hypertrophy was present in PDM patients even in the pain-free phase. In addition, the mediator role of the hypertrophic amygdala indicates the potential target of amygdala for anxiety treatment in PDM treatment in the pain-free phase.

**Supplementary Information:**

The online version contains supplementary material available at 10.1007/s11682-022-00664-3.

## Introduction

Primary dysmenorrhea (PDM) is characterized as painful, spasmodic cramping in the lower abdomen, without pelvic pathology, and is a common gynecological condition that affects most women throughout the menstrual years (Coco, 1999; Iacovides et al., 2015). Dysmenorrheic pain influence both the quality of life and the affective states because pain is not only is a sensory experience, but also an emotional event (Iacovides et al., 2015). Previous studies have manifested that the menstrual symptoms are associated with mood changes, including stress, depression and anxiety (Alonso & Coe, 2001; Balık et al., 2014; Dorn et al., 2009). The most prevalent conditions (e.g., anxiety disorders, and anxiety) can also increase dysmenorrhea and menstrual cycle problems(Burnett & Lemyre, 2017). The pathogenesis of these comorbidities is unclear despite the high association between mood disorder and PDM.

The current knowledge on PDM pathogenesis is the overproduction of uterine prostaglandins, which causes the uterine hypercontractility, reduces uterine blood flow, and increases peripheral nerve hypersensitivity-induced pain (Dawood, 2006; Iacovides et al., 2015). As a genuine type of chronic pain, recent neuroimaging studies have indicated that the brain structural and functional alteration is also involved in the pathophysiological of PDM (Low et al., 2018). The brain functional alteration in PDM patients has been found in descending pain modulatory systems and default mode network (Wei et al., 2016; Wu et al., 2016; Yu et al., 2021). For the structural brain, the abnormalities have been found in the regional gray matter volume (Tu et al., 2010, 2013) and cortical thickness (Liu et al., 2016), mainly involved in pain perception, transmission, modulation, as well as emotion processing (Low et al., 2018; Tu et al., 2013; Yang et al., 2019). For the subcortical volume, previous studies found decreased gray matter volume of caudate, thalamus and amygdala volume, and increased gray matter volume of the parahippocampal gyrus, and hypothalamus in patients with PDM (Liu et al., 2016; Tu et al., 2010). In addition, the brain structural and functional alterations are associated with pain intensity in patients with PDM. However, the subcortical shape alteration and its clinical significance in PDM were not explored hitherto.

FMRIB’s Integrated Registration and Segmentation Tool (FIRST) is a new automated structural MRI analysis tool in the FMRIB Software Library (FSL) for the subcortical nuclei segment. FIRST can achieve an individual-level segmentation of the outer surface of the substructure nuclei (Patenaude et al., 2011). FIRST segmentation of the amygdala is comparable with expert manual segmentation and performs better in the smaller subcortical structures in a scan–rescan (Morey et al., 2009, 2010). Importantly, FIRST could examine local changes in the subcortical nuclei. More recently, the shape abnormalities of subcortical brain structures have been considered to be potentially contributed to an in-vivo predictor of the neuropathological of disease (Gutman et al., 2022; Makkinejad et al., 2021). Thus, it is a useful tool for the subcortical morphometric analysis for neural mechanism studies of neuropsychiatric disease, like insomnia, addiction, Huntington’s disease and Parkinson’s disease (Gong et al., 2019a, b; Seifert et al., 2015; van den Bogaard et al., 2011). However, no study has investigated the subcortical shape alteration and its association with emotional features in the patients with PDM. Accordingly, a great deal of work is still to be done to elucidate the relationship between the altered subcortical structure and affective symptoms in the patients with PDM.

In the present study, we aimed to investigate the surface alterations of subcortical structures in patients with PDM by using FIRST combined with the shape analysis approaches. In addition, the correlation analysis was employed to explore the potential clinical significance of these alterations because the amygdala is the core region involved in emotion (Gallagher & Chiba, 1996), and its alteration has been manifested in PDM (Yang et al., 2019). Further, the mediation analysis was used to detect the underlying mediator role of the altered amygdala shape in the clinical relationships. We hypothesized that the amygdala shape alteration would be found in patients with PDM, and the altered amygdala shape characteristic may be associated with anxiety symptoms in PDM patients.

## Materials and methods

### Participants

Forty-five PDM patients were recruited mainly from the outpatient and inpatient sections of the gynecology department. Additionally, some PDM patients and 40 healthy controls (HC) were recruited from local communities and university campuses through flyer and social media advertisements (by YSY, WW and LLL). The study was approved by the Institutional Review Board of the Affiliated Hospital of Chengdu University of Traditional Chinese Medicine (No. 2013KL-033). All participants signed the informed consent. The inclusion criteria for PDM were as follows: The inclusion criteria for PDM is (1) a regular menstrual cycle (27–32 days); (2) Meeting the diagnostic criteria of primary dysmenorrhea under the Primary Dysmenorrhea Consensus Guideline (Burnett & Lemyre, 2017); (3) a history of PDM > 1 year (3–13 years, mean 5.46 years); (4) no exogenous hormones or centrally acting medication in the previous 6 months; (5) the cramping pain during menstruation in the previous 6-months should be rated > 4 by using visual analogue scale (VAS) (Larroy, 2002); and (6) right-handedness, as confirmed by the Edinburgh Handedness Inventory (RC, 1971). The exclusion criteria for PDM were: (1) suffering from other chronic pain conditions, like low back pain; (2) organic pelvic disease or abnormal found in the gynecological ultrasonography; (3) visceral pain and other neurology that may cause hyperalgesia; (4) positive pregnancy test or plan for pregnancy; (5) a history of neurological or psychiatric disorder history; (6) and having any contraindications for MRI scanning. The inclusion criteria for HC were: (1) a regular menstrual cycle (27–32 days); (2) the cramping pain measured by VAS during menstruation should be rated lower than 4; (3) normal neurological examination. The exclusion criteria of HC were similar to PDM.

## Clinical evaluation and biochemical test

Clinical and behavioral information was evaluated on the first 2 days of menstruation. The average pain severity of the last two menstrual stages was measured using the 0–10 visual analogue scale (VAS) from “no pain at all” to “unbearable pain (Larroy, 2002). The Zung Self-Rating Anxiety Scale (SAS) and Zung Self-Rating Depression Scale (SDS) were applied as secondary outcomes, in order to evaluate the anxiety and depression levels of PDM patients (Zung, 1971; Zung et al., 1965). In addition, Pain Catastrophizing Scale (PCS) was used to measure pain catastrophizing, which defined as an exaggerated negative response to imagined pain or actual pain (Meints et al., 2019).

Blood samples were collected in the morning on the first or next day of menstruation, and the serum levels of prostaglandin E2 (PGE_2_) and prostaglandin F2α (PGF_2α_) serum levels were measured using the Enzyme-Linked Immunosorbent Assay (ELISA) method.

## Imaging acquisition

All participants underwent MRI scanning on the same 3.0-Tesla magnetic resonance scanner (Discovery MR750, General Electric, Milwaukee, WI, USA) during the patients’ periovulatory phase (the middle five days between the two menstrual periods). Sagittal 3D T1-weighted images were acquired using a brain volume sequence with the following parameters: repetition time (TR) = 8.16 ms, echo time (TE) = 3.18 ms, flip angle (FA) = 7°, field of view (FOV) = 256 × 256 mm^2^, matrix = 256 × 256; slice thickness = 1 mm, voxel size = 1 × 1 × 1 mm^3^. no gap; and sagittal slices = 188.

## Image preprocessing

All the MRI data analyses were performed using the tools from FSL (version 5.0.9, https://fsl.fmrib.ox.ac.uk/fsl; FMRIB Software Library, Oxford University, Oxford, UK) (Jenkinson et al., 2012). This approach is based on Bayesian statistical models; the shape and appearance of subcortical structures are constructed from 336 manually labeled brain images provided by the Center for Morphometric Analysis, Massachusetts General Hospital, Boston. First, the SIENAX (https://fsl.fmrib.ox.ac.uk/fsl/fslwiki/SIENA) was used to estimate the total intracranial volume (eTIV), white matter volume (WM), and gray matter volume (GM) for all subjects; Second, the subcortical structures were segmented by using the FMRIB’s Integrated Registration and Segmentation Tool (FIRST, https://fsl.fmrib.ox.ac.uk/fsl/fslwiki/FIRST, part of FSL, version 5.0.9)(Patenaude et al., 2011). FIRST is an automated tool to segment the subcortical nuclei, and it has been used in several neuropsychiatry disorders studies (Gong et al., 2019a, b; Seifert et al., 2015; van den Bogaard et al., 2011); Third, the segmentation quality for each subject was checked manually (first_roi_slicesdir), after the automated segmentation (fun_first_all), and two patients with PDM were excluded in the next analysis after segmentation checking. Thus, data of 43 PDM patients were keep for all statistical analyses. The outcome file of FIRST was then used for volume and vertex analyses. All reported brain volumes were normalized to a “normalised” skull size (Smith et al., 2002). According to previous structural findings and our hypothesis, the volumes and shape alteration in the amygdala and hippocampus were analyzed in the current study.

## Surface-based shape analysis

The new version vertex-wise analysis was employed to investigate localized shape differences in the bilateral amygdala, which was adjusted for age and eTIV (first_utils and randomise, FSL 5.0.9). This approach calculated the group differences on a per-vertex basis, and controlled the effect of age and eTIV volume. The multiple comparison correction was used the Threshold-Free Cluster Enhancement (TFCE), a new method for finding significant “clusters” in the statistic image without having to define clusters in a binary way (Smith & Nichols, 2009). The traditional surface-based vertex analysis contained vectors in each significant vertex and was used it to display the direction of group differences.

Group differences on bilateral hippocampus were also calculated similarly because the hippocampus was the most reported abnormal subcortical structure in PDM (Tu et al., 2010).

### Statistical analysis

Statistical analysis was conducted with SPSS software version 20.0 (SPSS, Inc., Chicago, IL, USA). Two-sample t-test was employed to conduct the group difference of demographic, clinical symptoms, experimental test and subcortical volumes between the PDM and HC groups. In addition, the relationships between clinical features were detected using bivariate Pearson correlation analysis.

The mean shape value of the group differences on subcortical volumes was extracted for correlation analyses to further detect the potential associations between altered amygdala morphology and clinical experimental features (VAS, PCS, SAS, SDS scores, and PGE_2_ (PGF_2α_)) in the PDM group. The partial bivariate correlation analyses were then employed to detect the potential relationship between the altered amygdala and clinical features, adjusted for age and the eTIV. In addition, the significance level was set at *P* < 0.05, and false discovery rate (FDR) method was used for multiple comparison corrections.

## Mediation analysis

Significant associations between duration of disease and anxiety symptoms (SAS score) and catastrophizing (PCS score) were observed in PDM patients. The mediation analyses were then performed to examine whether the shape of the amygdala could serve as a potential mediator of these relationships. A simple mediation model from PROCESS Macro in SPSS was used for the mediation analysis (model 4) (Hayes, 2013). Age and eTIV were set as covariates. The detail of the mediation analysis can be found in a previous study (Yu et al., 2020), which was simply based on 10,000 bootstrap samples for a bias-corrected bootstrap confidence interval (CI). The indirect effect is considered significant when the 95% CI does not include zero (with a null hypothesis showing no indirect effect). The mediation analysis results were not corrected for multiple comparisons because sample size was small.

## Results

### Demographic information and clinical features

Table 1 shows that no significant group differences were found regarding in age, SDS, and PGF_2α_ between the PDM and HC groups (*P* > 0.05). SAS score, PCS score, and serum PGE_2_ were higher in patients with PDM compared to HC group (*P* < 0.05). The duration of disease was positively associated with anxiety symptoms (SAS score, *R =* 0.31, *P =* 0.04), catastrophizing (PCS score, *R =* 0.34, *P =* 0.03) in the PDM group. However, these relationships would not be significant after FDR correction. The anxiety symptom was positively associated with depressive symptoms (SDS score, *R =* 0.80, *P* < 0.001), and the serum PGE_2_ level was positively associated with PGF_2α_ in PDM patients (*R =* 0.71, *P* < 0.001). These relationships were also significant after FDR correction (**Table S1**).

**Table 1:** **Demographic, clinical characteristics and brain volume for two groups**

Characteristic	PDM (n = 43)	HC (n = 40)	*T* value	*p* value
Age	22.86 ± 1.98	23.42 ± 2.41	1.16	0.25
Duration (years)	5.46 ± 3.23	-	-	-
VAS	6.77 ± 1.53	0.18 ± 0.59	25.40	< 0.001
SDS	41.09 ± 12.75	36.43 ± 11.17	1.76	0.08
SAS	41.09 ± 12.75	33.84 ± 7.47	3.41	0.001
PCS	11.76 ± 12.77	1.85 ± 5.04	4.59	< 0.001
PGE_2_ (µg/L)	361.00 ± 156.23	275.70 ± 128.97	2.54	0.01
PGF_2α_ (µg/L)	175.23 ± 71.92	166.63 ± 90.99	0.46	0.65
Gy matter volume	808.16 ± 33.26	812.35 ± 34.24	0.56	0.57
White matter volume	689.58 ± 26.26	688.81 ± 34.24	0.21	0.83
estimate total intracranial volume	1497.75 ± 448.44	1500.49 ± 553.98	0.35	0.80
Left Amygdala volume	0.99 ± 0.21	1.01 ± 0.21	0.15	0.68
Right Amygdala volume	0.96 ± 0.25	1.01 ± 0.29	0.91	0.36
Left Hippocampus volume	3.72 ± 0.37	3.68 ± 0.41	0.47	0.64
Right Hippocampus volume	3.92 ± 0.39	3.88 ± 0.40	0.42	0.67

Abbreviations: PDM, primary dysmenorrhea; HC, healthy control; VAS, visual analogue scale; SDS, self-rating depression scale; SAS, self-rating anxiety scale; PCS, pain catastrophizing scale; PGE_2_, Prostaglandin E2; PGF_2α_, Prostaglandin F2α

## Brain volume alteration in patients with PDM

The eTIV, GM and WM volumes were not significantly different between the two groups. Moreover, no significant difference in volume between the PDM and HC groups was noted for the bilateral amygdala and hippocampus (Table 1). The Partial correlation analyses revealed that no significant associations exist between subcortical volumes (bilateral amygdala and hippocampus) and clinical features in the PDM group (*P* > 0.05, **Table S1**).

## Amygdala shape alteration in patients with PDM

**Fig. 1:**
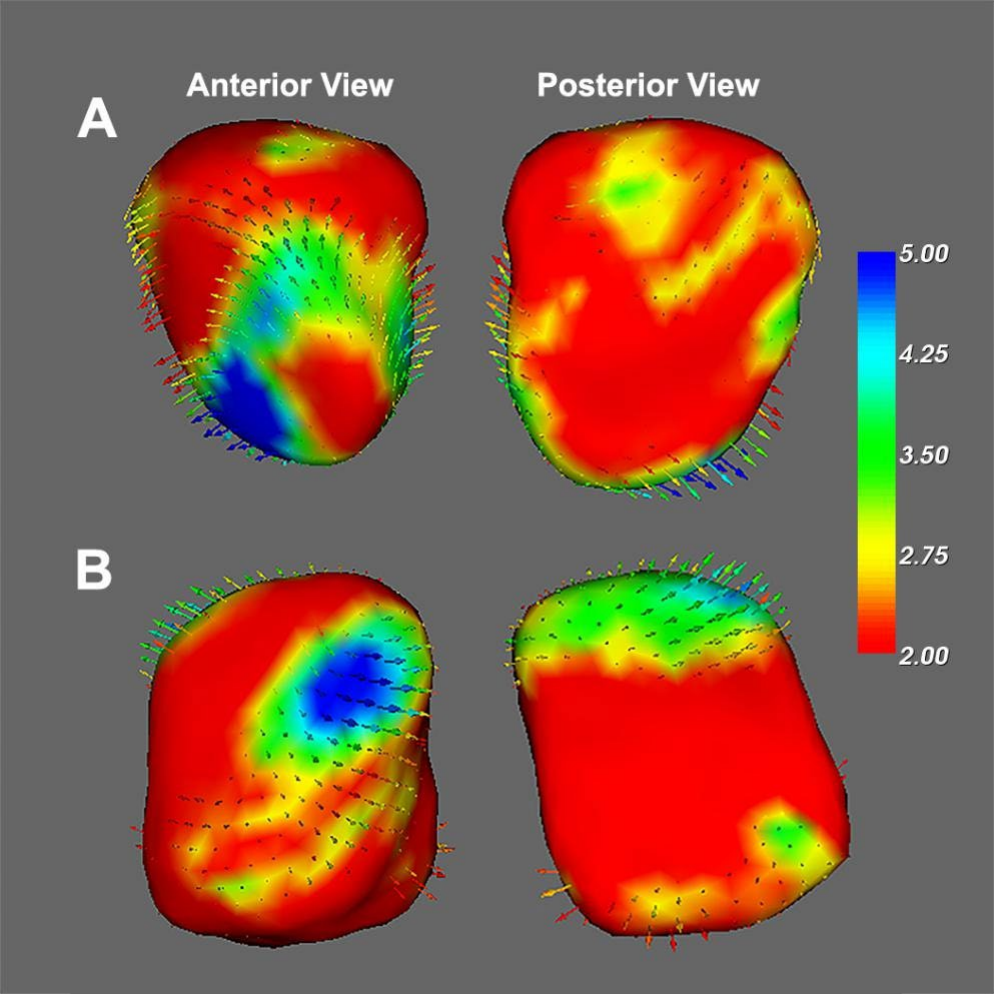
**The localized shape differences between the HC and PDM groups using vertex-wise surface analyses of the amygdala.***Orang*e regions indicate the part of the amygdala shown to be abnormal in patients with PDM. (**A**) The group difference in the left amygdala was located in the superficial nuclei of the amygdala. (**B**) The group difference in the right amygdala was located in the superficial nuclei and basolateral nuclei of the amygdala

**Fig. 2:**
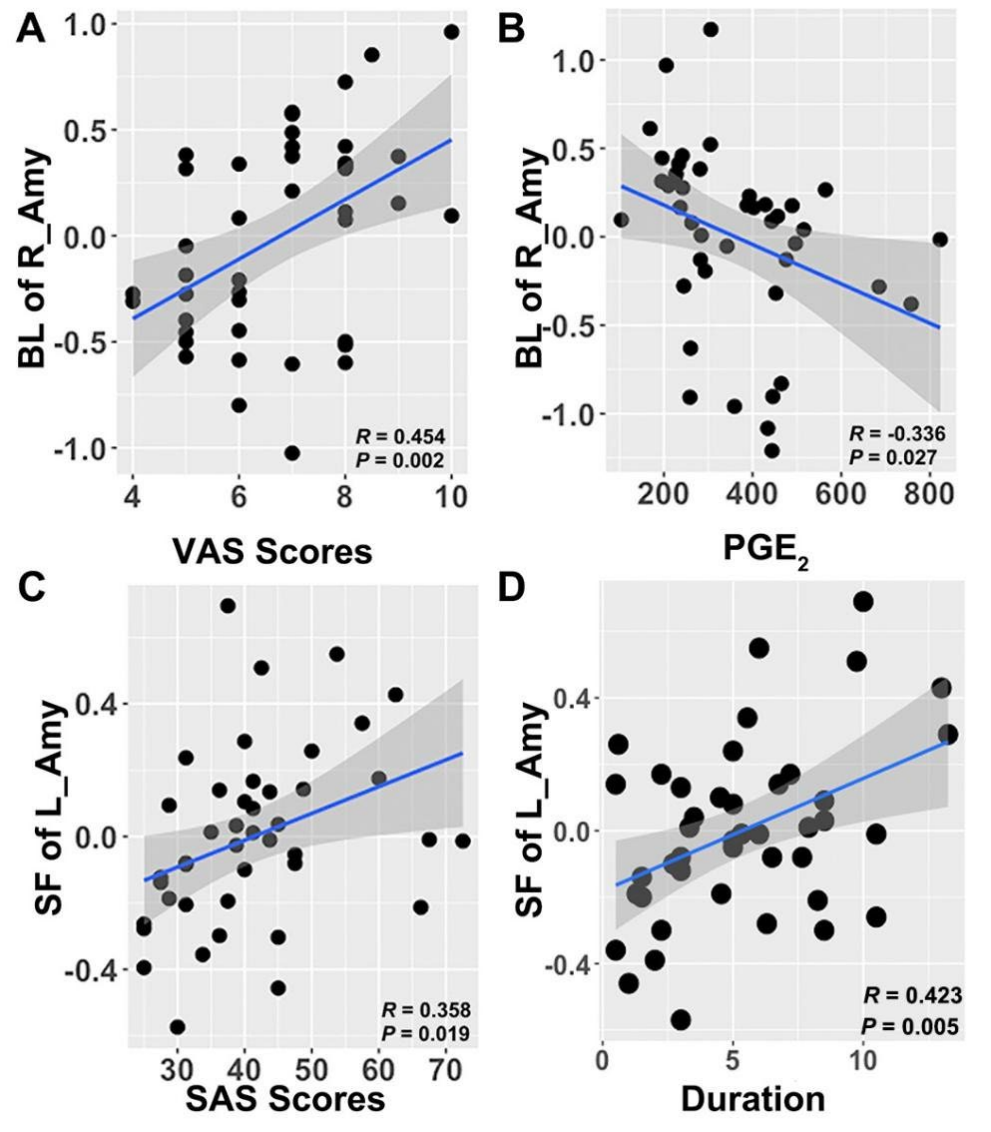
**Vector graphs of the amygdala according to the traditional surface-based vertex analysis displayed by the 3D mesh. A** is left amygdala; **B** is right amygdala. The color bar indicates the *T* value. The *arrows on the surface* indicate the direction of change. The *arrows outward the surface* indicate the direction of the difference showing that the amygdala is expansive here compared with the healthy control group

As shown in Fig. 1, the vertex-based shape analysis revealed that the superficial nuclei of the left amygdala, and the superficial and basolateral nuclei of the right amygdala showed significant group differences in the PDM compared with the HC group (using TFCE correct approach). The traditional surface-based vertex analysis showed the outward displacement in these significantly different regions of the amygdala (Fig. 2), and the findings of shape analysis indicated that the localized amygdala volume was expansive in the PDM group compared to the HC group. In addition, no significant areas of atrophy were observed in PDM patients compared to the HC group. Furthermore, no significant group difference was found in the shape analysis of the bilateral hippocampus.

## Clinical significance of shape alteration in PDM patients

**Fig. 3:**
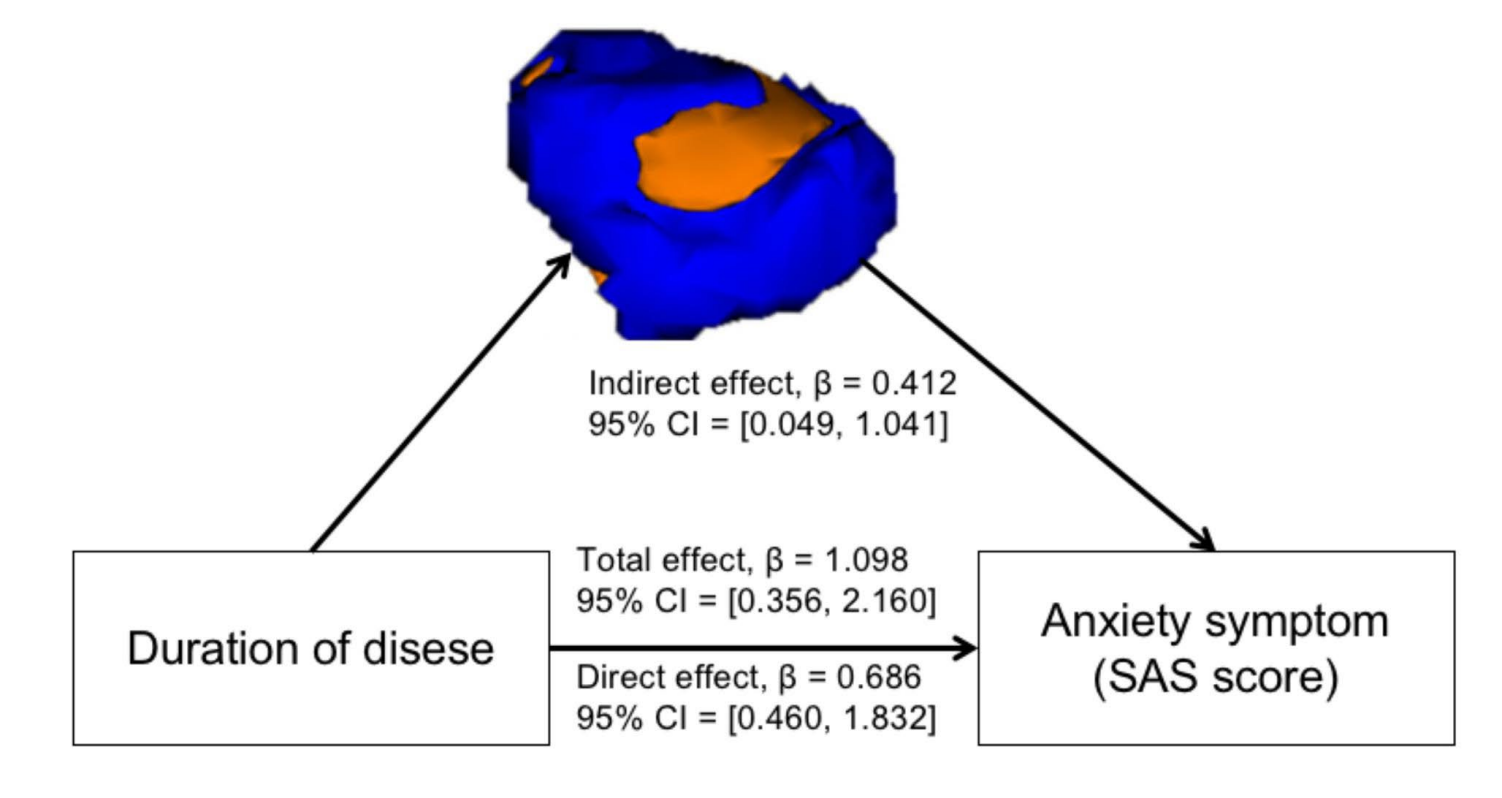
**The clinical significance of the altered shape of the amygdala in the PDM group. A** the altered shape of the BL of the right amygdala is positively correlated with VAS score in PDM; **B** the altered shape of the BL of the right amygdala is negatively correlated with the serum level of PEG_2_ in PDM group; **C** and **D** the altered shape of the SF of the left amygdala is positively correlated with SAS score and disease duration in PDM; Abbreviations: PDM, primary dysmenorrhea; BL, basolateral; SF, superficial; VAS, visual analogue scale; PGE_2_, Prostaglandin E2; SAS, self-rating anxiety scale

The potential clinical associations of the altered amygdala morphology in the PDM group were detected using partial correlation analysis. Figure 3 shows that the VAS score was positively associated with the hypertrophic basolateral nuclei of the right amygdala (*R =* 0.454, *P =* 0.002), the serum level of PGE_2_ was negatively correlated with the hypertrophic basolateral nuclei of the right amygdala (*R =* -0.336, *P =* 0.027), and the disease of duration and SAS score were positively associated with the hypertrophic superficial nuclei of the left amygdala (duration, *R =* 0.423, *P =* 0.005; SAS, *R =* 0.358, *P =* 0.019) in the PDM group. The relationship between the basolateral nuclei of the right amygdala and VAS as well as the relationship between the superficial nuclei of the left amygdala and duration were also significant after FDR correction. No significant association was found between altered amygdala shape and clinical features.

## Hypertrophic left amygdala mediated the association between duration and anxiety severity and catastrophizing

**Fig. 4:**
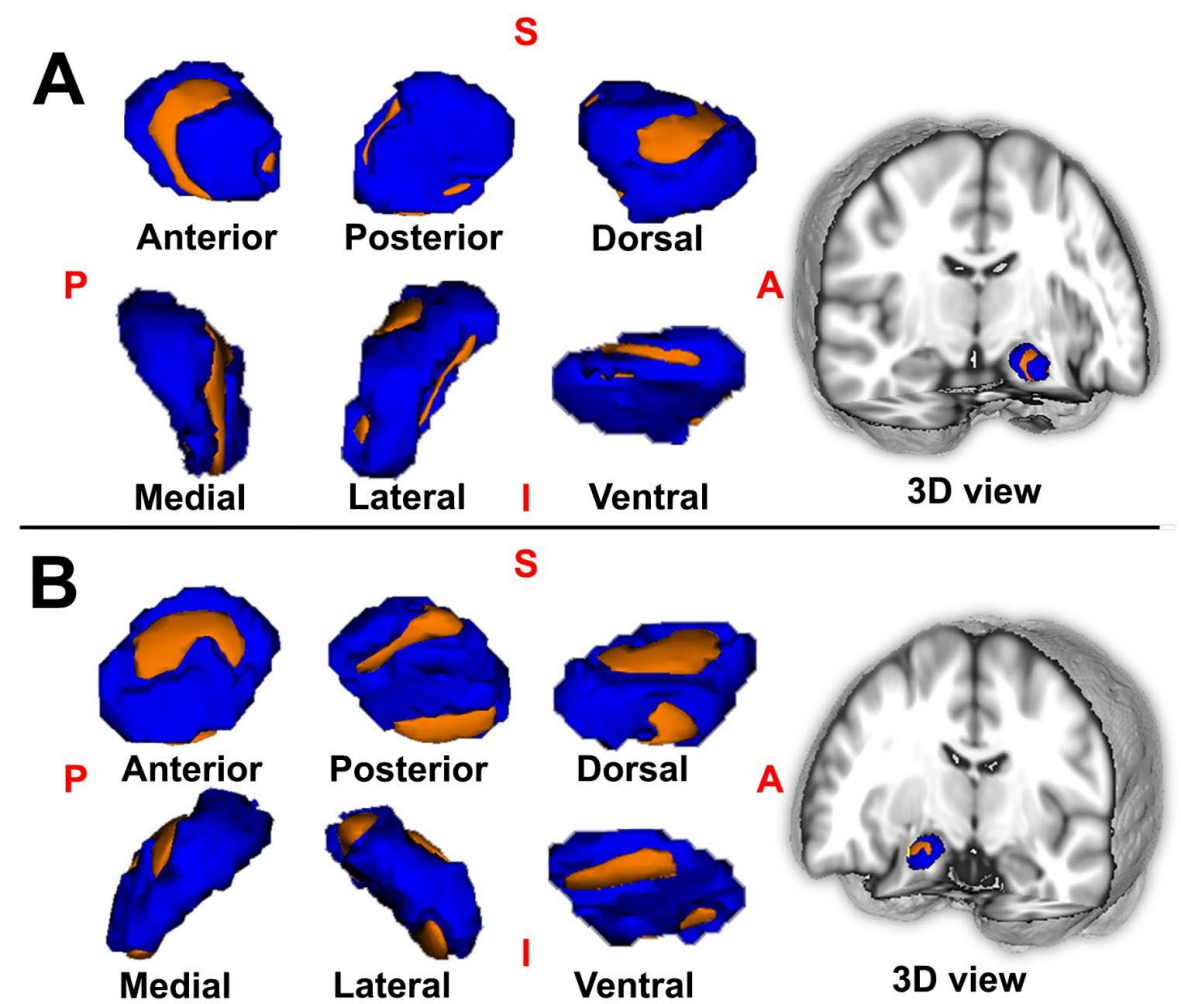
**Hypertrophic left amygdala mediated the association between duration and anxiety severity in PDM patients.** Abbreviations: PDM, primary dysmenorrhea; SAS, self-rating anxiety scale

Correlation analysis has confirmed the positive relationship between duration and anxiety (SAS score) in patients with PDM (*R =* 0.31, *P =* 0.04). The hypothesized mediation effect of the shape of the altered amygdala shape on the relationship between duration of disease and anxiety in PDM was then tested. Mediation analysis revealed a significant indirect effect of the superficial of left amygdala on the relationship between disease duration and anxiety in patients with PDM during the menstrual phase (*β* = 0.412, 95% CI = 0.049, 1.041) (Fig. 4), the results indicating that the hypertrophic superficial of left amygdala mediated the effect of disease duration on anxiety symptoms. Moreover, no mediation effects were found for other altered amygdala shapes.

As the clinical association between duration and catastrophizing (PCS score) was found in PDM group. The mediation analysis was also conducted to estimate the mediation effect of the shape of the altered amygdala shape on the relationship between duration of disease and anxiety in PDM. However, no significant indirect effect was found in the mediation analysis.

## Discussion

This study is believed to be the first to explore the morphological alteration in the amygdala and its potential relationship with pain severity and mood symptoms in patients with PDM. Three main findings were presented in the study: (1) the hypertrophic changes were found in the bilateral amygdala in the pain-free phase in PDM, but not the whole volume of the amygdala. The left hypertrophic amygdala was located in the superficial nuclei, whereas the right hypertrophic amygdala was located in the superficial and basolateral nuclei in the patients with PDM; (2) the hypertrophic amygdala in the pain-free phase was associated with both disease duration, pain severity and anxiety symptoms in the menstrual phase in PDM; and (3) the hypertrophic superficial of the left amygdala could mediate the disease duration- associated anxiety severity in PDM. Taken together, the results indicated the regional hypertrophic amygdala in the pain-free phase in PDM. Moreover, the mediator of the hypertrophic amygdala in the association between duration and anxiety will help elucidate the neural mechanisms underlying the anxiety disorder in the PDM patients.

Shape analysis of subcortical nuclei is a new approach to detect the potential structural alterations in neuropsychiatry diseases, rarely implemented in PDM research. The volumetric analysis did not show any significant group difference between the PDM and HC groups, and the results are similar to the finding of a previous structural study using voxel-based morphometry (VBM) analysis in the periovulatory phase of PDM (Yang et al., 2019). However, the regional alteration in the amygdala was detected by shape analysis, indicating that the overall volume estimate may not capture the structural alteration in patients with PDM during the periovulatory phase. Thus, the current study proposed that the shape analysis could be a useful tool to detect the subcortical alterations in PDM in the pain-free stage. Anatomically, the amygdala is composed of three subregions: basolateral nuclei, superficial nuclei, and centromedial nuclei (Amunts et al., 2005). Functionally, the basolateral nuclei are associated with sensory input, the superficial nuclei are particularly sensitive to social and emotional information processing, and the centromedial nuclei are linked to attention mediation and response preparation (Bzdok et al., 2013). The results of the current study showed that the hypertrophic amygdala was located in the bilateral superficial and right basolateral nuclei, indicating a maladaptive mechanism of the amygdala in the sensory input and social information processing during the pain-free stage in PDM patients.

The analysis of the current study also confirmed the association between hypertrophic amygdala during the pain-free stage and disease duration as well as VAS during menstruation in PDM patients. The basolateral nuclei of the amygdala is a likely integrator of the preprocessed perceptual input, including visual, auditory and somatosensory information (Bzdok et al., 2013; Pessoa, 2010). In the present study, the hypertrophic basolateral right amygdala was associated with pain severity and PGE_2_ serum level in PDM menstrual phase. Thus, the results of the current study indicated that the shape of the basolateral amygdala may contribute to pain information prediction before the pain input during the menstrual phase. In addition, the hypertrophic superficial amygdala was associated with disease duration and anxiety symptoms during menstruation in PDM. The superficial amygdala was thought to play a decisive role in social interaction, such as social hierarchy assessment and reproductive behavior in mammals and higher primates (Dulac & Torello, 2003; Winans & Scalia, 1970). Moreover, resent resting-state functional connectivity analysis has found that the superficial amygdala is functionally connected with the anterior cingulate cortex and limbic lobe (Roy et al., 2009), which contributes to fear, negative affect, and pain (Shackman et al., 2011). Previously, the shape of the amygdala have been reported associated with anxiety symptoms in chronic insomnia (Gong et al., 2019a, b). The present findings of the associations between anxiety, duration, and hypertrophic superficial amygdala may indicate the PDM patient’s fear of the social interaction with others during the menstrual phase. Thus, the hypertrophic amygdala during the pain-free stage would be indicated the compensatory mechanism in the menstrual cycle in PDM patients.

The hypertrophic superficial amygdala could be interestingly a mediator in the disease duration-associated anxiety symptoms in PDM during the menstrual phase because the superficial amygdala is mainly involved in social information integration (Bzdok et al., 2013). This mediation effect of hypertrophic superficial amygdala indicated the social-related stress model of anxiety symptoms in PDM (Wang et al., 2004). PDM is a leading cause of absenteeism, thus, the patients with PDM suffer many social stresses before the menstrual phase (Sahin et al., 2018), and the anxiety would be combined during the pain-free stage (Stewart & Deb, 2014; Yamada et al., 2019). The result implicates that the social stress related alteration of the amygdala contributes to the disease duration related anxiety during the pain-free stage. According to this model, the current study proposed that the superficial amygdala would be a target for preventing anxiety during the menstrual phase. Thus, the social stress should be released in cognitive-behavioral therapy during the pain-free stage.

Pain catastrophizing, which defined as an exaggerated negative response to imagined pain or actual pain, have been reported increased in patients with PDM, especially the first day of the menstrual cycle (Cosic et al., 2013). Previous researches reported the catastrophizing was associated pain severity in women with dysmenorrhea (Evans et al., 2021). The present study did not find the association between catastrophizing and pain severity in PDM, but the pain catastrophizing associated with duration of disease. The different results might attribute to the different inclusion criteria of dysmenorrhea. Previous neuroimaging studies have indicated that the sensor cortex (S1), anterior insula, dorsolateral prefrontal cortex and cingulate cortex was involved in the processing of pain catastrophizing (Galambos et al., 2019). The present study suggest that the amygdala shape might not involve in pain catastrophizing in patients with PDM.

The present study has several limitations. First, this was a cross-sectional study, and how the hypertrophic amygdala mediates the duration associated anxiety symptoms in PDM over time is unclear. A longitudinal design study may help to resolve this question. Second, as PDM is a cyclic chronic pain, the present MRI scan is during the pain-free phase. Recently, Yang et al. have found the larger amygdala volume in patients with patients with PDM during the menstrual phase but not the periovulatory phase by using VBM analysis (Yang et al., 2019). Thus, future studies should also explore the amygdala shape alteration during the periovulatory phase in PDM. Third, the effects of age and eTIV were regressed as confounding factors. But previous studies reported that age has little effect on amygdala and hippocampus volumes (Saygin et al., 2015; Uematsu et al., 2012), whereas the global correction using total brain volume provides greater specificity and sensitivity than total intracranial volume in elderly adult (Bigler & Tate, 2001). In our study, the average age is 22.86, with the age range from 20 to 28. Thus, future studies would verify our findings in elderly patients with PDM. Forth, the sample size of the current study is relatively small, the mediation analysis was based on the disease of duration associated anxiety symptom, which was not significant after multiple comparisons, and the mediation analysis results were also not corrected for multiple comparisons. Thus, future studies with larger sample sizes and multiple sites are needed to validate the results of the current study.

## Conclusions

The results of the current study demonstrated that localized amygdala shape hypertrophy was present in PDM patients even in the absence of pain. In addition, the mediator role of the hypertrophic amygdala in the disease duration-associated anxiety may indicate the potential target for PDM treatment before the menstrual phase.

## Electronic Supplementary Material

Below is the link to the electronic supplementary material.

Supplementary Material 1

Supplementary Material 2

## Data Availability

Not applicable.
