# Supplementary material for: The hypertrophic amygdala shape associated with anxiety in patients with primary dysmenorrhea during pain-free phase: insight from surface-based shape analysis
Source: Brain Imaging Behav. 2022 Jul 24;16(5):1954–63. doi: 10.1007/s11682-022-00664-3 (PMC9581870; doi:10.1007/s11682-022-00664-3)
Supplement: Supplementary file 1 — Supplementary Material 1 [file 11682_2022_664_MOESM1_ESM.docx]

Table S1. The relationships between clinical features and brain volumes in PDM group

| Items | Statistical values | Duration | VAS | SAS | SDS | PCS | PGE_2_ | PGF_2α_ | L_Hip | R_Hip | L_Amy | R_Amy |
| --- | --- | --- | --- | --- | --- | --- | --- | --- | --- | --- | --- | --- |
| Duration | R | 1.00 | 0.21 | 0.31* | 0.11 | 0.34* | 0.11 | 0.22 | 0.05 | -0.08 | -0.03 | -0.22 |
|  | P |  | 0.18 | 0.04 | 0.49 | 0.03 | 0.48 | 0.15 | 0.77 | 0.60 | 0.86 | 0.16 |
| VAS | R | 0.21 | 1.00 | 0.22 | 0.00 | 0.01 | -0.07 | 0.07 | -0.04 | 0.10 | -0.17 | -0.12 |
|  | P | 0.18 |  | 0.15 | 0.98 | 0.93 | 0.67 | 0.65 | 0.82 | 0.53 | 0.28 | 0.46 |
| SAS | R | 0.31* | 0.22 | 1.00 | 0.80** | 0.22 | 0.05 | 0.14 | 0.29 | -0.10 | -0.14 | -0.03 |
|  | P | 0.04 | 0.15 |  | 0.00 | 0.17 | 0.76 | 0.37 | 0.06 | 0.52 | 0.38 | 0.87 |
| SDS | R | 0.11 | 0.00 | 0.80** | 1.00 | 0.26 | -0.04 | 0.04 | 0.30 | -0.07 | -0.01 | 0.10 |
|  | P | 0.49 | 0.98 | 0.00 |  | 0.09 | 0.78 | 0.81 | 0.06 | 0.64 | 0.95 | 0.54 |
| PCS | R | 0.34* | 0.01 | 0.22 | 0.26 | 1.00 | 0.29 | 0.13 | 0.21 | 0.14 | 0.01 | -0.11 |
|  | P | 0.03 | 0.93 | 0.17 | 0.09 |  | 0.06 | 0.42 | 0.19 | 0.37 | 0.98 | 0.49 |
| PGE_2_ | R | 0.11 | -0.07 | 0.05 | -0.04 | 0.29 | 1.00 | 0.71** | 0.00 | -0.24 | 0.06 | -0.05 |
|  | P | 0.48 | 0.67 | 0.76 | 0.78 | 0.06 |  | 0.00 | 1.00 | 0.12 | 0.70 | 0.75 |
| PGF_2α_ | R | 0.22 | 0.07 | 0.14 | 0.04 | 0.13 | 0.71** | 1.00 | 0.01 | -0.26 | -0.10 | -0.09 |
|  | P | 0.15 | 0.65 | 0.37 | 0.81 | 0.42 | 0.00 |  | 0.93 | 0.09 | 0.52 | 0.57 |
| L_Hipp | R | 0.05 | -0.04 | 0.29 | 0.30 | 0.21 | 0.00 | 0.01 | 1.00 | 0.05 | -0.27 | -0.02 |
|  | P | 0.77 | 0.82 | 0.06 | 0.06 | 0.19 | 1.00 | 0.93 |  | 0.74 | 0.08 | 0.90 |
| R_Hipp | R | -0.08 | 0.10 | -0.10 | -0.07 | 0.14 | -0.24 | -0.26 | 0.05 | 1.00 | 0.03 | 0.15 |
|  | P | 0.60 | 0.53 | 0.52 | 0.64 | 0.37 | 0.12 | 0.09 | 0.74 |  | 0.85 | 0.35 |
| L_Amy | R | -0.03 | -0.17 | -0.14 | -0.01 | 0.01 | 0.06 | -0.10 | -0.27 | 0.03 | 1.00 | 0.65** |
|  | P | 0.86 | 0.28 | 0.38 | 0.95 | 0.98 | 0.70 | 0.52 | 0.08 | 0.85 |  | 0.00 |
| R_Amy | R | -0.22 | -0.12 | -0.03 | 0.10 | -0.11 | -0.05 | -0.09 | -0.02 | 0.15 | 0.65** | 1.00 |
|  | P | 0.16 | 0.46 | 0.87 | 0.54 | 0.49 | 0.75 | 0.57 | 0.90 | 0.35 | 0.00 |  |

Note: ^*^ *P*<0.05, ^**^, *P*<0.001. Abbreviations: PDM, primary dysmenorrhea; VAS, visual analogue scale; SDS, self-rating depression scale; SAS, self-rating anxiety scale; PCS, pain catastrophizing scale; PGE2, Prostaglandin E2; PGF2α, Prostaglandin F2α; L_Hip, left hippocampus volume; R_Hip, right hippocampus volume; L_Amy, left amygdala volume; R_Amy, right amygdala volume.
